# Determining the Possibilities of Reducing Visible Quality Defects in Commercial Elements of Turkey Carcasses Using an Alternative Stunning Device

**DOI:** 10.3390/foods12173141

**Published:** 2023-08-22

**Authors:** Joanna Katarzyna Banach, Ryszard Żywica, Małgorzata Grzywińska-Rąpca, Mariola Grzybowska-Brzezińska

**Affiliations:** 1 Institute of Management and Quality Sciences, Faculty of Economics, University of Warmia and Mazury in Olsztyn, 10-719 Olsztyn, Poland; ryszard.zywica@uwm.edu.pl; 2 Department of Market and Consumption, Institute of Economics and Finance, Faculty of Economics, University of Warmia and Mazury in Olsztyn, 10-720 Olsztyn, Poland; malgo@uwm.edu.pl (M.G.-R.); margrzyb@uwm.edu.pl (M.G.-B.)

**Keywords:** turkey meat, electrical water-bath stunning, current frequency, quality defects, hemorrhages

## Abstract

The development of the quality and commercial value of poultry meat is related to the formation of visible quality defects (hemorrhages) in muscles during the first stage of production (stunning). The production of meat with an unusual appearance for the consumers affects their purchasing decisions and, consequently, the company’s economics. The aim of this research was to determine the possibility of reducing visible quality defects (large and small hemorrhages) in commercial turkey carcass elements (fillet, loin, wing) using an alternative device (AD) for the electrical stunning of animals in comparison to the quality effects obtained using the conventional device (CD) in plant X. The factors differentiating the experiment were the electrical current frequency (AD: 125, 400, 800, and 1600 Hz; CD: 50 Hz) and process time (9 and 18 s). The increase in electrical current frequency used in the alternative device stunner (own construction) resulted in changing the percentage share of defective turkey meat production. The greatest reduction of minor and severe meat defects and improvement of its quality were obtained for the alternative device at f = 800 Hz and t = 9 s—considered optimal for specific industrial conditions. Extending the time of stunning turkeys to 18 s had a positive effect on visible quality defects in the evaluated commercial elements of the carcass; however, its application in practice will depend on the efficiency of the slaughter line of the plant. A comparative analysis of the results of the impact of the frequency of electric current in the alternative device and plant X on the improvement of meat quality showed a justified need to commercialize the research results and replace the device currently used in the plant with an alternative one.

## 1. Introduction

The increased awareness of meat producers regarding factors and processes that induce defects of poultry meat has urged the management of processing plants to undertake actions aimed at meat quality improvement. Diminished meat quality and commercial value of poultry meat are mainly related to the occurrence of meat quality defects in the form of extravasations—hemorrhages [1,2]. They are irreversible and difficult to eliminate defects, constituting up to 20% of defects in a batch of slaughtered poultry. This defect also bears some economic consequences to processing plants, which are due to both internal deficits, such as losses in deliveries, excision of hemorrhages, and non-compliance with quality standards, and external deficits. According to scientists [3,4,5], the reason behind the development of this irreversible quality defect of meat is the process of electrical stunning in a water bath. Despite this finding, it is still the method of choice in Poland and in most countries worldwide. Bearing in mind that the high level of quality costs debilitates competitiveness of enterprises on the market, the reduction in quality costs is sought in innovative solutions, which are expected to facilitate slaughter procedures and to enable the production of high-quality meat [6,7].

Despite the innovative actions undertaken, scientists are still conducting research on improving the efficiency of existing slaughter lines and optimizing the electrical parameters of stunning devices for animals. It has been demonstrated that the use of low frequencies of electrical current (50 and 100 Hz) in the stunning process of turkeys contributed to an increase in the number of hemorrhages and bone fractures, whereas the use of higher frequencies (500–1500 Hz) contributed to faster bleeding, a lower number of bone fractures, and of resultant extravasations on the skin and in pectoral major muscles [8,9,10]. When investigating the effect of the choice of electrical current parameters on the extent of animal stunning, Girasole et al. [11] and Smaldone et al. [5] concluded that the maximum probability of successful stunning may be achieved with high stunning frequencies, which induce a lower occurrence of lesions on carcasses; however, on the other hand, these require greater current intensities to be effective. Research by EFSA scientists [1] conducted in this respect have formed grounds for the development of Council Regulation (EC) 1099/2009 [12]—which specified for different bird (chickens, turkeys, ducks, and geese) species not only the values of the current (I) but also the frequency (f) ranges, which ensure the welfare of birds at slaughter and the high quality of their meat. For turkeys, the values of these parameters were determined as follows: f < 200 Hz and I = 250 mA; f = 200–400 Hz and I = 400 Hz; f = 400–1500 Hz and I = 400 mA. Moreover, the duration of electrical stunning is also specified for animals—it must be at least 4 s, but the choice of the duration still depends on the yield of a slaughter line in a given processing plant [13,14].

Considering the ambiguous character of the effects of technical and technological parameters of the water bath stunning process on the quality of poultry meat, research was carried out to determine the possibility of reducing visible quality defects (large and small hemorrhages) in commercial elements of turkey carcasses (fillet, loin, wing) using an alternative device for stunning animals compared to the qualitative effects obtained using a conventional device in plant X. The factors differentiating the experiment were the frequency of the current used in devices (50, 125, 400, 800, and 1600 Hz) and the process time (9 and 18 s). The aim of this research was also to indicate the optimal parameters of an alternative device for electrical stunning of turkeys, guaranteeing high-quality meat.

## 2. Materials and Methods

### 2.1. Slaughter and Meat Acquisition

**Figure 1:**
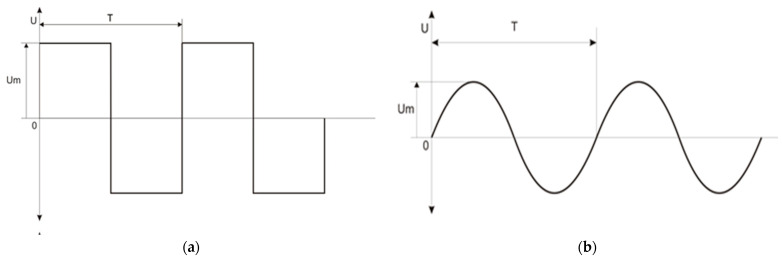
Characteristics of the alternating current (AC) used in the device: alternative (**a**) and conventional—plant X (**b**), where: T = full period time, Um = peak voltage.

Experimental material included heavy-type turkey toms type BIG-6. The birds were transported in cages with vehicles from production farms to a slaughterhouse in northeastern Poland. To ensure a comparative experimental design at each stage of the study, the birds subjected to experimental and control slaughter originated from the same supplier and were unloaded manually from the same vehicle. The industrial process of stunning in water-bath, supervised by the veterinarian, was carried out on-line using:

➢ The alternative device, AD [15,16]—rectangular wave AC (Figure 1a) and four tested frequencies of electric current (f): 125, 400, 800 and 1600 Hz.
➢ The conventional device, CD (plant X equipment)—manufactured in The Netherlands: sine wave AC (Figure 1b), f = 50 Hz.

The current values (250 and 400 mA) used during the research were in accordance with the requirements of Council Regulation (EC) 1099/2009 [12]. The alternating current voltage in both devices varied in the range of 325 to 337 V. Another factor that differentiated the research was the time of stunning: 9 s—standard and 18 s—extended.

The study on the impact of an alternative device for stunning turkeys on the reduction of quality defects (large and small hemorrhages) of meat was carried out on the automatic slaughter line (yield 1000 birds/h) in two stages:

✓ Stage I, optimization of stun parameters using an alternative device: frequency (f)—125, 400, 800, and 1600 Hz for 9 and 18 s. Raw material was from supplier 1 (good quality).
✓ Stage II, comparison of stunning using two devices: alternative device (the most effective parameters from stage I, f = 800 Hz) and conventional device, f = 50 Hz. Raw material came from supplier 2 (low quality).

The quality of the supplied raw material was classified on the basis of the company’s quality assessment protocol for live turkeys, in which the percentage of birds that died during transport, the degree of their plumage, and weight diversity were estimated.

At each stage—for each device, frequency, and time—the research was carried out on a batch of 100 birds. Turkey carcasses were air-chilled with water spray (air temperature 2–3 °C, time 24 h, air flow rate 1 m/s, periodic spraying of the carcass with water) and subjected to industrial cutting for the culinary parts ca. 24 h after slaughter.

### 2.2. Evaluation of Visible Quality Defects of Carcass Elements

Meat quality defects were evaluated by a five-person panel on 30 of 100 randomly selected commercially valuable parts of carcasses, i.e., m. pectoralis major (fillet), m. pectoralis minor (loin), and m. biceps brachii (wing). The methodology of quantitative evaluation of quality defects in meat were developed, taking into consideration some quality problems occurring in the slaughterhouse. The evaluation of the meat quality defects was based on two main criteria: (1) small hemorrhages, ø < 19 mm—minor defects (MD) and (2) large hemorrhages, ø > 20 mm—severe defects (SD). Meat defect was defined as the percentage of meat with small or large hemorrhages. Such an evaluation enabled us to determine the high-quality (premium) meat rate—expected by consumers [17,18].

### 2.3. Data Analysis Methods

Main effects ANOVA were used to assess the influence of traits (carcass parts, current frequency and duration of stunning, type of device) on the dependent variable (hemorrhages: small and large).

## 3. Results and Discussion

### 3.1. Stage I—Optimization of Stunning Parameters by Alternative Device

The basis for the implementation of the first stage of the research was literature data and the results of our own research [19,20], which show that the factors affecting the occurrence of quality defects (hemorrhages) on the commercial elements of the carcass include, among others, the inappropriate selection of electric current parameters in the process of water bath electric stunning. Therefore, the process of optimizing the frequency of the alternative current of the device for stunning birds and the duration of this process was carried out in the first place. These activities made it possible to determine the level of reductions of visible meat quality defects and justify taking preventive actions as part of improving integrated quality management systems and production control [21].

The results of the evaluation of quality defects of meat from turkey toms stunned with an alternative device for 9 s showed that the use of f = 125 Hz affected the occurrence of minor quality defects (small hemorrhages) in the majority of the evaluated elements (97%). The greatest degree of their occurrence (73%) was observed in the wings. In loins and fillets, minor defects were present at the level of 47%. Severe defects (large hemorrhages) were observed in 50% of the evaluated meat elements. The most severe quality defects were again observed in wings (33%), fillets (30%), and the least in loins (17%)—Table 1.

The use of f = 400 Hz resulted in the appearance of the highest percentage of minor (MD) and severe defects (SD) of meat in wings and fillets, which was MD: 30 and 20% and SD: 23 and 15%, respectively. The smallest defects were found in the loins (3%) with only small hemorrhages (less than 20 mm), and large hemorrhages were not observed. In addition, the extension of stunning time of the turkeys had a negative impact on the occurrence of minor quality defects (MD) in wings—40%, and loins—23%. The smallest defects (MD—7%) were found in fillets. Considering the above, it was found that increasing the frequency (125–400 Hz) in the alternative device affected the reduction of minor and serious defects in meat, in general, and in the individual carcass meat elements (Table 1).

The application of f = 800 Hz resulted in the occurrence of the largest minor and severe quality defects in wing muscles (33 and 13%) and fillets (17 and 10%). With the extended time of stunning (18 s), the share of small and large hemorrhages in the evaluated elements was lower than when using the standard time (9 s). In wings, it was 20 and 10%, respectively, and in fillets, 13 and 7%. It was also observed that the time of stunning had no effect on the defectiveness of loins, which are the most delicate and the most expensive commercial elements of poultry meat. A comparison of the evaluation of meat quality defects conducted for turkeys stunned with an electrical current used at the frequencies of f = 400 Hz and f = 800 Hz shows that increasing the frequency in this range resulted in a decrease in the percentage share of assessed carcass elements with severe quality defects, especially after stunning for 9 s (Table 1). It is worth emphasizing that using f = 800 Hz resulted in the greatest quality improvement in fillets, which are considered to be the distinguishing feature of the quality of meat offered on the market (in terms of price and nutritional value).

The application of a higher current frequency (f = 1600 Hz) for 9 s resulted in an increase in the production of defective carcass elements compared to the quality of meat produced using f = 400 Hz and f = 800 Hz. The evaluation of meat defects (minor—MD and severe—SD) indicated that the frequency of 1600 Hz had the most adverse effect on loin defects (MD—55%, SD—40%) and fillets (MD—20%, SD—15%), which are the most commercially valuable. Comparing the impact of stunning using an alternative device (own construction) at f = 400 Hz and f = 1600 Hz for 9 s, it was observed that the MD of fillets, regardless of the size of hemorrhages, was at a similar level of 20%, but SD was slightly lower—15%. In the wings, both minor and severe qualitative defects were at the level of 15%—Table 1.

The elongation of stunning time from 9 to 18 s had a positive effect on meat quality defects; however, the application of such a long stunning procedure is feasible only in processing plants with a low-yield slaughter line [13,14]. In addition, this phenomenon contradicts the observations made by Schütt-Abraham and Wormuth [22] and Hindle et al. [23], who claimed that the elongation of stunning time cannot compensate for misadjusted electrical parameters.

**Figure 2:**
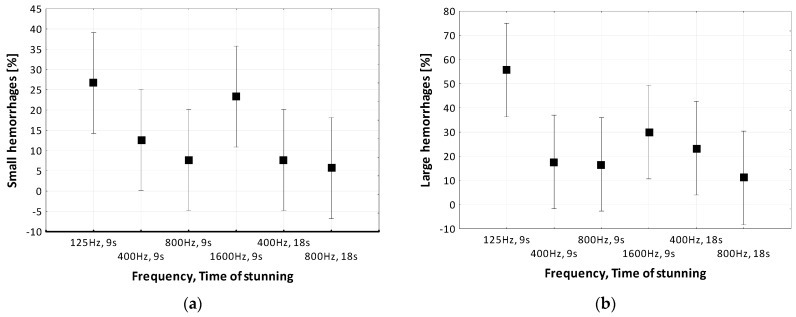
Average values of (**a**) small and (**b**) large hemorrhages in the examined elements of the meat carcass, taking into account the parameters of the stunning process (current frequency, time).

Statistical analysis was performed for two groups: minor quality defects—small hemorrhages (Figure 2a), and severe quality defects—large hemorrhages (Figure 2b). Based on the results of Levene’s test, showing the homogeneity of variance for groups 1 and 2 (*p* = 0.25 and *p* = 0.21 is > α = 0.05), it was assumed that the variances in the studied groups are homogeneous. This means that the mean values of small hemorrhages (Figure 2a) occurring at 400 Hz and 800 Hz for 9 s differ significantly from the mean values at f = 125 Hz and f = 1600 Hz for 9 s and at 400 Hz and 800 Hz for 16 s. In the case of large hemorrhages (Figure 2b), their average values at f = 125 Hz and f = 1600 Hz differ significantly from the values at other frequencies of the stunning parameters.

The results of the analysis of variance (Table 2) confirmed the earlier observations (Table 1) that the parameters of stunning had a significant (α = 0.05) effect on the occurrence of small hemorrhages in meat, while the occurrence of large hemorrhages was slightly less affected by this factor (*p* ≈ 0.10). The qualitative factor—elements of the carcass—however, did not significantly affect the occurrence of minor and severe quality defects (*p* = 0.22, *p* = 0.28).

Results of measurements performed at the first stage of the study demonstrated that the lower percentage of visible quality defects (minor and severe) of commercial elements of meat carcasses was achieved upon the use of the frequency of f = 800 Hz, whereas the greatest one—upon the use of f = 125 Hz (Table 1). Obtained trends in meat quality improvement (decrease in the production of defective meat) were also confirmed in their research on broiler and turkey meat [19,24]. This beneficial effect of f = 800 Hz on meat quality by Mouchoniere et al. [25,26] was explained by proper stunning and more efficient bleeding instead.

### 3.2. StageII—Effectiveness Comparison: Alternative and Conventional Devices

Considering that in the first stage the greatest reduction of meat quality defects was demonstrated with the use of an alternative device with f = 800 Hz, the statical analysis confirmed the significant impact of the current frequency on the occurrence of hemorrhages. The second stage compared the impact of two types of devices, alternative and commercial, on the occurrence of these defects in meat obtained from low-quality raw material (from supplier 2). Taking into account the aspect of the type of device and the value of low and high frequencies of the electrical current allowed us to determine their impact on the efficiency of high-quality meat production (without defects) in the research.

The research carried out in this area showed that the elements of carcasses of turkeys stunned using a conventional device with f = 50 Hz were characterized by the greatest defects. Most minor defects were observed in loins (90%) and in wings (65%), while severe defects (difficult to remove and costly) were also found in loins (40%). In fillets and wings, SD defects were at the levels of 20 and 15%, respectively. The use of f = 800 Hz in an alternative device (indicated in the first stage as a parameter affecting the improvement of meat quality) also confirmed the occurrence of meat defects at the lowest level compared to the defects occurring with the conventional device (f = 50 Hz). The least severe quality defects for manufacturers, in the form of large hemorrhages, occurred with the use of the alternative device. The level of defectiveness of the loin produced was 25%, and in the case of fillets and wings, it was only 10% (Table 3).

**Figure 3:**
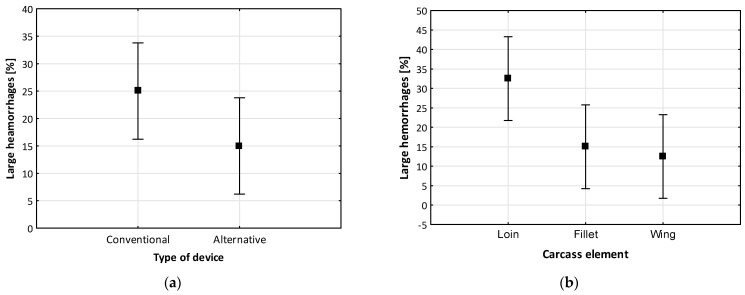
Average values of large hemorrhages, taking into account type of device (**a**) and carcass element (**b**).

The analysis of the homogeneity of variance for two groups of quality defects—hemorrhages (*p* = 0.39 and *p* = 0.37 > α = 0.05) in relation to the type of device and elements of the carcass were authorized by the Levene significance test. The obtained results of statistical calculations (Table 4) showed that at the level of *p* ≈ 0.10, the type of device and, thus, the parameters used in them (f = 50 Hz, f = 800 Hz) affected the occurrence of both large (Figure 3a) and small hemorrhages.

A greater impact on the occurrence of visible quality defects had the elements of meat carcasses than the type of device. A significant effect (*p* = 0.05) of this factor was obtained in relation to the occurrence of large hemorrhages in meat—Figure 3b. In the case of small hemorrhages, it was lower (*p* = 0.11) but comparable to the level of influence of the type of device. The mean values of large hemorrhages in fillets and wings differed significantly from the values in loins (Figure 3b). Data presented in the categorized Figure 3a, taking into account the type of devices (conventional and alternative), confirm significant differences between the average values of large hemorrhages.

The analysis of the literature data indicates that the occurrence of a large share of defective elements of turkey carcasses subjected to stunning using a device with f = 50 Hz was probably caused by cardiac arrest of the birds; insufficient bleeding of the carcasses; and, consequently, the occurrence of a large number of hemorrhages in the wings, on the fillet cross-section, and in the tenderloin [25,26]. Banach [20] and Huang et al. [27], on the other hand, explained the mechanism of the electric current frequency on muscle tissue on the basis of structural changes in muscle tissue. The current of f = 50 Hz flows selectively, taking the path of least resistance (mainly through the circulatory system) and going through the places of vital contusions, causing the formation of hemorrhages. The current of f = 800 Hz, flowing evenly through the whole body of the turkey, causes a much greater degradation of the structure of the muscle tissue and has a positive effect on the tenderness of the meat and its defectiveness.

Sabow et al. [28] defines the practical aspects of using these two frequencies. Stunning with low-frequency electrical current in a water bath, due to the presence of quality defects, as well as its non-compliance with halal rules, is criticized. In plants with low production capacity, devices that use high frequency are perceived more favorably. The reasons for this are the preservation of animal welfare, efficient bleeding and improvement of carcass/meat quality, and the possibility of reversible stunning (after approx. 2 min, the bird could regain consciousness), which meets the minimum requirements for halal slaughter [29]. With such preferences, plant employees select the frequency value on their own, which often results in discrepancies in its effectiveness on the quality and welfare of animals. In addition, in the current Council Directive EC 1099/2009 [12], the parameters of electric current (frequency, intensity) of stunning devices according to Lines et al. [6] and Sirri et al. [30] affect the deterioration of meat quality—the occurrence of severe quality defects (large hemorrhages).

The observed higher level of meat defects in stage II compared to stage I could be caused by the lower quality of the raw material supplied to the plant, thus contributing to the increased occurrence of quality defects in the form of bruises and hemorrhages in the meat [5,19,20]. Despite the still high variation in the quality of the raw material delivered to the poultry plant (conditions and time of transport, plumage, weight variation) and the quality problems of the meat produced [18], an alternative device, by increasing the share of high-quality meat (without hemorrhages) by about 50%, can meet consumers’ expectations regarding the visual features of its cut elements [31,32]. It can also reduce technological losses [4], increase the production of high-quality culinary meat [20], and be one of the tools for building the competitive advantage of companies [32,33].

## 4. Conclusions and Applications

1. The greatest reduction of severe and minor meat defects and improvement of its visual quality was obtained for the alternative device at f = 800 Hz and t = 9 s—considered optimal for specific industrial conditions.
2. The double elongation of turkey stunning procedure time had a positive effect on the percentage of quality defects observed in the evaluated elements; however, the use of such a long time in the stunning process in practice is strictly connected with the lower efficiency of the plant’s slaughter line.
3. A comparative analysis of the results of the impact of the frequency of the electric current used in the alternative and conventional device on the improvement of meat quality showed a justified need to commercialize the research results and replace the device currently used in the plant with an alternative one.
4. The implementation of an alternative device will allow manufacturers to take preventive measures and will also indirectly improve its technological features (shelf-life, color), economic indicators (quality costs, production efficiency), and the natural environment (waste).

## Figures and Tables

**Table 1:** Percentage of minor (MD) and severe (SD) quality defects (small and large hemorrhages) of meat of turkey toms stunned with alternative device stunner at the frequency of 125, 400, 800, and 1600 Hz during 9 and 18 s (*n* = 30).

Quality Defects	f [Hz]	Meat Quality Defects [%]			
		Total	Wing	Loin	Fillet
Time of stunning, 9 s					
MD	125	97	73	47	47
	400	47	30	3	20
	800	47	33	0	17
	1600	65	15	55	20
SD	125	50	33	17	30
	400	30	23	0	15
	800	23	13	0	10
	1600	50	15	40	15
Time of stunning, 18 s					
MD	400	50	40	23	7
	800	33	20	0	13
SD	400	23	20	0	3
	800	17	10	0	7

**Table 2:** Results of the analysis of variance between a group of meat defects (small and large hemorrhages) and quality factors—stunning parameters and carcass elements.

Quality Factors	SS	DF	MS	F	*p*
Small hemorrhages					
Stunning parameters	3852.94	5	770.59	3.40	0.05
Carcass elements	804.11	2	402.06	1.78	0.22
Large hemorrhages					
Stunning parameters	1196.94	5	239.39	2.55	0.10
Carcass elements	274.11	2	137.06	1.46	0.28

Explanations: SS—inter-group variance; MS—intra-group variance, DF—degrees of freedom, F—Snedecor’s F distribution, *p*—significance level.

**Table 3:** Percentage of quality defects, minor (MD) and severe (SD), of meat of turkey toms stunned with the conventional and alternative devices.

Quality Defects	Type of Device	Meat Quality Defects [%]		
		Loin (*n* = 30)	Fillet (*n* = 30)	Wing (*n* = 30)
MD	Conventional	90	25	65
	Alternative	50	15	25
SD	Conventional	40	20	15
	Alternative	25	10	10

**Table 4:** Results of the analysis of variance between meat defects (small and large hemorrhages) and factors—type of device, elements of the carcass meat.

Qualitative Factor	SS	DF	MS	F	*p*
Small hemorrhages					
Carcass element	2500.00	2	1250.00	8.33	0.11
Type of device	1350.00	1	1350.00	9.00	0.10
Large hemorrhages					
Carcass element	475.00	2	237.50	19.00	0.05
Type of device	150.00	1	150.00	12.00	0.07

Explanations: SS—inter-group variance; MS—intra-group variance, DF—degrees of freedom, F—Snedecor’s F distribution, *p*—significance level.

## Data Availability

The data presented in this study.
